# A Preliminary Study of the Effect of DBD Plasma and Osmolytes on T98G Brain Cancer and HEK Non-Malignant Cells

**DOI:** 10.3390/molecules18054917

**Published:** 2013-04-25

**Authors:** Nagendra Kumar Kaushik, Pankaj Attri, Neha Kaushik, Eun Ha Choi

**Affiliations:** Plasma Bioscience Research Center, Kwangwoon University, Seoul 139701, Korea; E-Mails: chem.pankaj@gmail.com (P.A.); neha.bioplasma@gmail.com (N.K.)

**Keywords:** osmolytes, plasma, brain cancer cells, non-malignant cells

## Abstract

Non-thermal plasmas are emerging as a novel tool for the treatment of living tissues for biological and medical purpose. In this study, we described the effect of 4 min dielectric barrier discharge (DBD) plasma on both T98G cancer and HEK normal cell lines in the presence of different concentrations of osmolytes. This treatment strategy shows a specific inhibitory effect of a 240 s plasma exposure in the presence of osmolytes against T98G brain cancer cells only, but not on HEK normal cells. Based on these interesting properties of osmolytes, a non-thermal plasma appears to be a potential anticancer treatment strategy for different kinds of cancers in the presence of osmolytes.

## 1. Introduction

The non-thermal plasma (an ionized gas) is emerging as a novel tool for the treatment of living tissues for biological and medical purposes [1,2,3,4,5,6,7,8,9,10,11,12,13,14,15,16,17,18,19,20,21]. The plasma is considered as the fourth state of matter and is obtained by using a supply of energy. This excited gas contains free charges (electrons, ions), free radicals, excited molecules and photons (UV) and generates transient electric fields. Several previous studies have demonstrated the efficacy for sterilization by plasma application, whereas some studies have shown an antitumor effect of plasma *in vitro* on a few types of cancer cells [2,3,4,5,6]. Current research mainly focuses on the non-thermal effects of dielectric barrier discharge (DBD) and jet plasmas: applications below the threshold of thermal damage (slightly above room temperature) aim at inducing a specific response or chemical modification by generating active species that are either produced in the plasma or in the tissue brought into contact with plasma [7,8,9,10,11,12,13,14,15,16,17,18,19,20,21]. Non-thermal plasmas also have some effect on the healthy tissue or cells, but allow efficient biological activity (*i.e*., anticancer, antibacterial, antifungal activity and effect against other disorders/diseases) within a minute. After *in vitro* studies on various animal and human cells, the application of a cold atmospheric plasma to live tissue remains only a question of time. A broad spectrum of medical applications in healthcare, in particular the anticancer properties of cold plasmas, have paved the way for analyses on living human tissues and patients. Recently, we reported micronucleus formation in T98G cells by DBD plasma depends on plasma exposure and incubation time [20].

Nowadays, the main concern of this emergent field is to protect normal tissues from the effect of the plasma at the time of treatment. For this specific purpose, we used different concentration of osmolytes in our present study. Osmolytes have many unique protective metabolic roles, such as acting as antioxidants, providing redox balance, detoxifying sulfide, protecting macromolecules, enhancing protein folding and regulating cell volume [21,22,23,24,25,26,27,28]. There are many reports that osmolytes could protect proteins/enzymes from the different kinds of stress that cause denaturation [21,22,23,24,25].

Methylamines and polyhydric alcohols are two main classifications of osmolytes, which perform important roles in many protein folding studies. Trimethylamine N-oxide (TMAO) is the strongest natural occurring osmolyte and belongs to the methylamines family, whereas, glycerol and sucrose belong to the polyhydric alcohols. These osmolytes are natural occurring and they are present in human body, therefore these molecules act as protectants to human being. The basic mechanism is that these osmolyte chemical structures provide the preferential interactions between the surface of cells and neighboring particles of cosolvents through exclusion of osmolytes. This exclusion of osmolytes is due to the H-bonding that is formed between osmolytes and water [21,22,23,24,25].

**Figure 1:**
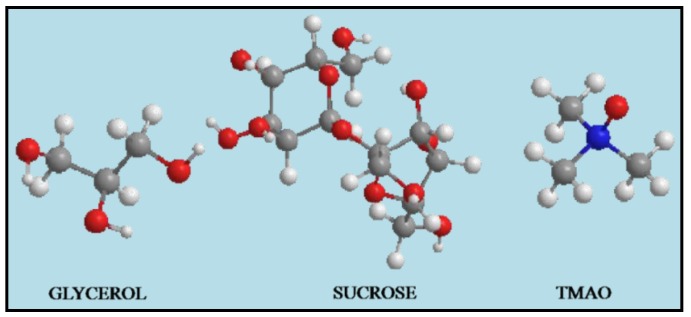
Energy minimized structure of osmolytes used in study. Brown, white, red and blue color balls represent carbon, hydrogen, oxygen, and nitrogen atoms, respectively.

By considering the unique behavior of these osmolytes, in this present study, we determine the synergistic effect of a DBD plasma and osmolyte on T98G brain cancer and HEK non-malignant cell lines. The HEK cell line resembles developing neuron and neuronal stem cells and are also mentioned as good model for neuroscience studies. Hence we have used HEK normal cells as a model for brain normal cells in this study. In this work we show the effect of a 240 s exposure of DBD plasma on normal HEK and T98G brain cancer cells in the presence of glycerol, TMAO and sucrose. The structures of osmolytes are displayed in Figure 1. The main goal of this study is to protect normal cells from the toxic effect of the plasma and to induce cell death in diseased cells.

## 2. Results and Discussion

**Figure 2:**
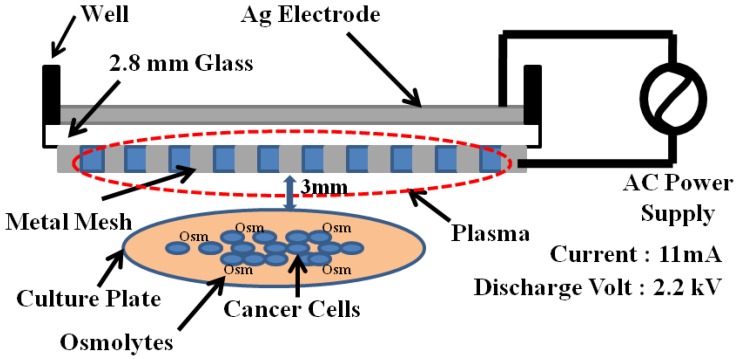
Schematic diagram of dielectric barrier discharge plasma system consisting mainly of a high-voltage power supply, electrodes, and dielectrics.

In order to test the effects of plasma treatment on mammalian cells in the presence of osmolytes, we applied a DBD plasma to T98G human brain cancer and HEK non-malignant cells. Our dielectric barrier discharge plasma system mainly consists of a high-voltage power supply, electrodes, and dielectrics (Figure 2). For the high-voltage power supply, a commercial transformer for neon light operated at 60 Hz is used [20]. The primary voltage of the high-voltage transformer by Slidacs is regulated by using voltage controller. The upper electrode is made of silver (Ag) and the lower electrode facing the sample is made of stainless-steel mesh. They are separated by 2.8 mm thick glass and tightly sealed by an insulating paste.

The diameter of the electrodes is 9 cm, which is designed for a 10 cm Petri dish. Plasmas were delivered from a source placed at a fixed distance of 3 mm above the media being treated; the plasma is released as an electrical discharge as a result of electrical breakdown of the air in the gap between the high-voltage electrode and the substrate being treated. We had divided treatment by osmolytes with or without plasma on cells in different groups (Table 1).

**Table 1:** Group description for treatment of human cells.

Group	Osmolytes (25, 50, 75 mM)	Plasma (min)
**1**	Glycerol	0
**2**	Glycerol	4
**3**	TMAO	0
**4**	TMAO	4
**5**	Sucrose	0
**6**	Sucrose	4
**7**	--	4

**Figure 3:**
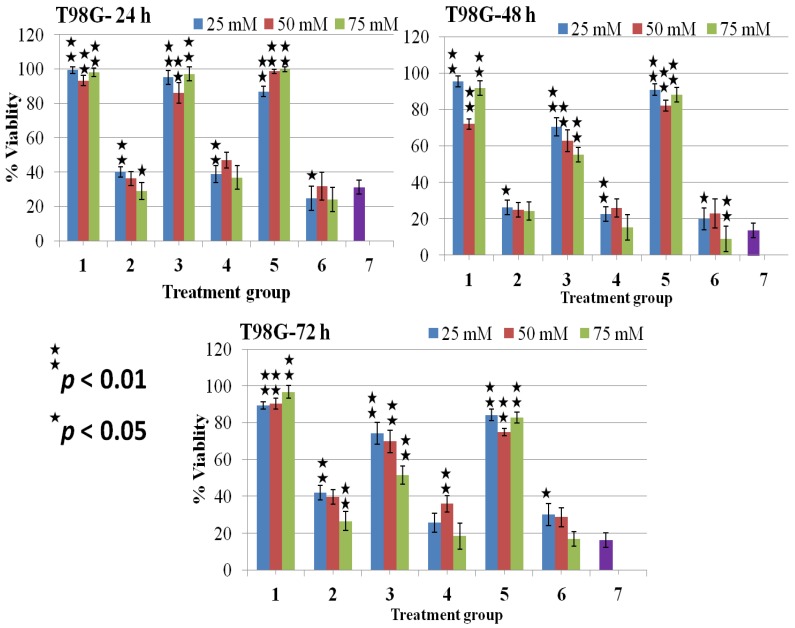
% Viability evaluated from MTT assay on treated T98G brain cancer cells at 24, 48, and 72 h. Data presented are mean ± SD of at least three independent experiments (n = 3) and statistically analyzed.

**Figure 4:**
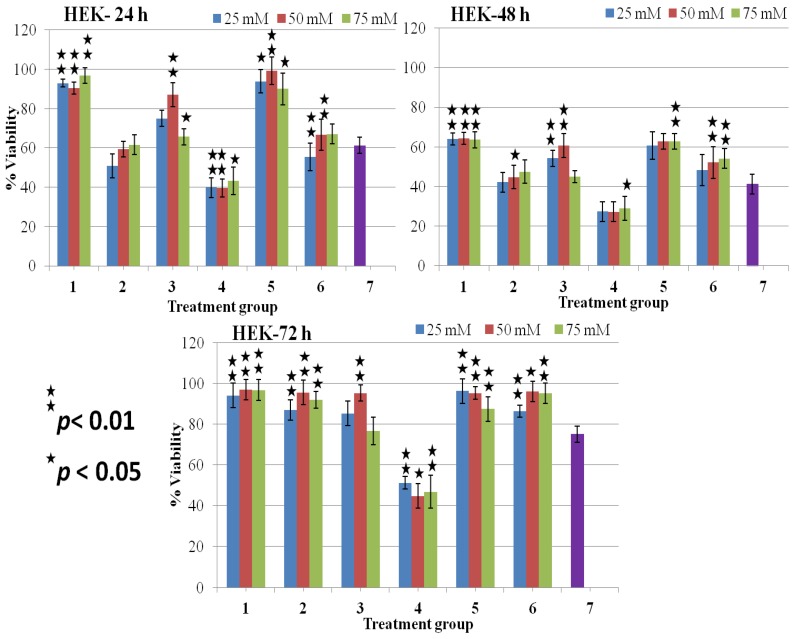
% Viability evaluated from MTT assay on treated HEK non-malignant cells at 24, 48, and 72 h. Data presented are mean ± SD of at least three independent experiments (n = 3) and statistically analyzed.

In the earlier work, we measured viability of T98G cells with time and there we found that after 240 s of DBD plasma treatment inhibited T98G cancer cells up to 70%–90% and maximum inhibition occurred at 72 h incubation after treatment [20]. Thus, this experiment was involved in establishing the concentration-dependent effects of osmolytes with or without a 240 s plasma treatment on cell viability by MTT assay. At doses 25–75 mM, the glycerol and the sucrose shows significant viability without plasma, which is more than 85% in both T98G and HEK cells (Figure 3, Figure 4). The decrease in cell viability depends on the concentration and the incubation time in the presence of TMAO without plasma (Figure 3). At higher concentration of TMAO, cell viability was in range of 44%–70% in both cancer and normal cell lines after 48 h of incubation (Figure 3, Figure 4). We observed that T98G cells treated with plasma (without osmolytes) showed a significant decrease in viability and that their range decreased to 13%–31%. However, the plasma showed less effect on HEK normal cells, and their viability range were more than 75% after 72 h of treatment (Figure 4). 

Plasma-treated T98G cancer cells in the presence of glycerol showed a concentration dependent decrease in cell viability, the maximum inhibitory effect was found after 48 h of treatment, and their range was 24%–39% (Figure 3). However, plasma-treated T98G cells in the presence of sucrose showing a 8.9%–30% viability, the maximum inhibitory effect was found at 48 h with 75 mM concentration (Figure 3). The plasma in presence of TMAO showed almost the same inhibitory effect as glycerol and sucrose on T98G cells, their viability range being 15%–40%. The maximum inhibitory effect was observed after 48 to 72 h of plasma treatment in the presence of all three osmolytes.

HEK normal cells treated with plasma in the presence of glycerol and sucrose showed an inhibitory effect up to 48 h after treatment, and their viability range was 50%–70% (Figure 4). However, HEK cells at 72 h showed significant viability more than 86% in the case of sucrose and glycerol (Table 2 and Figure 4). A decreased survival was observed in plasma-treated HEK cells in the presence of TMAO, showing almost the same cytotoxic effect as shown in T98G cells, and their viability range was is 27%–40% in HEK cells (Figure 4). Whereas, HEK cells showed some more viability at 72 h after treatment in the presence of glycerol and sucrose when compared with their counter plasma treated cells (Table 2 and Figure 4). Moreover, HEK treated cells in the presence of TMAO showed a decrease in cell viability, and the viability percentage was less than 50% when compared to the untreated control (Table 2 and Figure 4).

**Figure 5:**
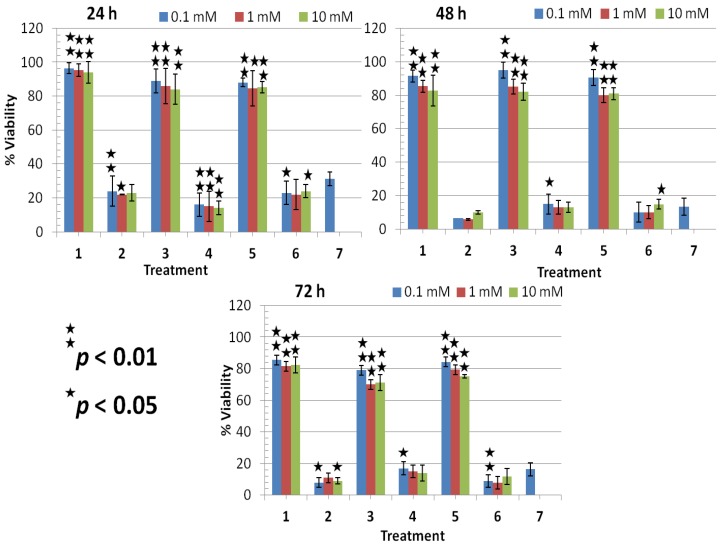
% Viability evaluated from MTT assay on treated T98G malignant cells at lower concentrations of osmolytes (0.1 mM, 1 mM and 10 mM) with or without plasma. Data presented are mean ± SD of at least three independent experiments (n = 3) and statistically analyzed.

We also measured viability of T98G and HEK cells at low concentrations of osmolytes (sucrose glycerol and TMAO). In this experiment low concentration of osmolytes were used with or without plasma treatment for 240 s on cell viability. At doses 0.1, 1 and 10 mM, the osmolytes without a plasma shows significant viability in both HEK and T98G cells, which is more than 80% and 75% respectively (Figure 5). 

**Table 2:** Percentage viability after 72 h of treatment. T98G cells were treated with a osmolytes at a 75 mM concentration with or without DBD plasma for 4 min. All values are means of three independent experiments.

Treatment	T98G (%)	HEK (%)
DBD Plasma (4 min)	16.29	75
Glycerol + Plasma	26.61	101.9
TMAO + Plasma	18.43	46.9
Sucrose + Plasma	16.82	95.2
Glycerol	96.76	96.74
TMAO	51.45	76.57
Sucrose	82.74	87.46

**Figure 6:**
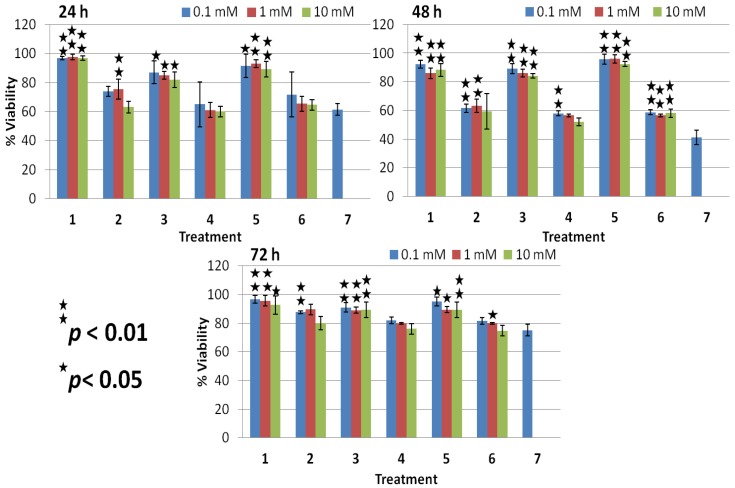
% Viability evaluated from MTT assay on treated HEK normal cells at lower concentrations of osmolytes (0.1 mM, 1 mM and 10 mM) with or without plasma. Data presented are mean ± SD of at least three independent experiments (n = 3) and statistically analyzed.

However, we found significant decrease in cell viability of T98G cells in case of osmolyte treatment with plasma at lower concentrations (0.1–10 mM). The decrease in cell viability of T98G cells depends on the incubation time in the presence of osmolytes with plasma. The maximum inhibitory effect was observed after 72 h of treatment, and their viability range was 8%–17% at 0.1–10 mM concentration (Figure 5). The maximum inhibitory effect was observed on T98G cells in the case of the plasma with osmolytes. While, HEK normal cells treated with plasma in the presence of low concentrations (0.1–10 mM) of osmolytes showed an less inhibitory effect up to 48 h of treatment, and their viability range was 60%–80% (Figure 6). However, HEK cells at 72 h showed significant viability and showed more than 76% or up to 96% viability or survival in the case of osmolytes at low concentrations (Figure 6).

**Figure 7:**
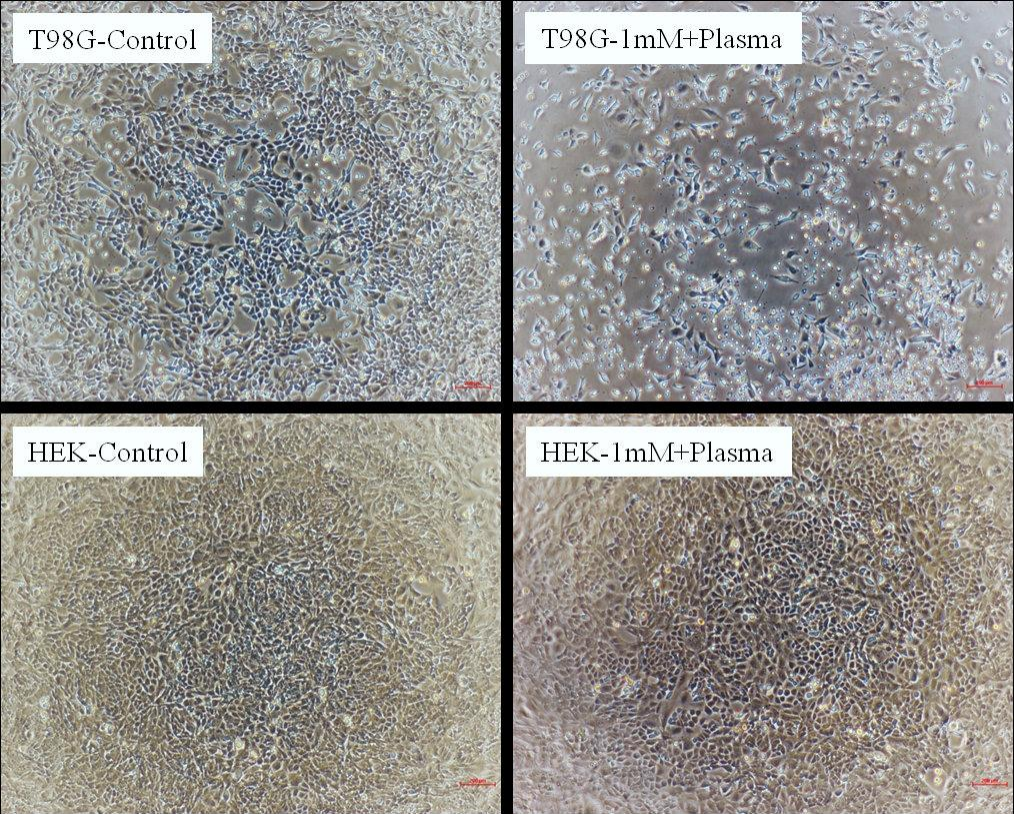
Morphology of T98G and HEK non-malignant cells after 72 h of treatment at 1 mM concentrations of sucrose with plasma (240 s). Control is untreated control cells. Images are taken by NIKON eclipse T*i* inverted microscope.

Whereas, 0.1 and 1 mM concentration of osmolytes (sucrose, glycerol and TMAO) showed significant increase in viability of HEK cells at all incubation time (24–72 h) after plasma treatment when compared with their counter only plasma (without osmolytes) treated cells (Figure 6, Figure 7 and Table 3).

Figure 7 shows the differences in the viability of a treated cells population as compared to the untreated cells. Cell morphology analysis revealed that the only T98G cells were affected by 1 mM sucrose with plasma exposure. This clearly shows that at low concentration osmolytes counteract the effect of plasma only on HEK non-malignant cells.

## 3. Experimental

### 3.1. General

MTT (3-[4,5-dimethylthiazol-2-yl]-2,5-diphenyltetrazolium bromide), *N*-[2-hydroxyethyl] piperazine-*N*-[2-ethanesulfonic acid] (HEPES) buffer, ribonuclease-A (RNase-A), and tris-hydrochloride were obtained from the Sigma Chemical Co. (Yongin, Korea). Trypsin-EDTA was obtained from Gibco (Grand Island, NY, USA). Antibiotic-antimycotic solution and phosphate buffer saline (PBS) were obtained from Welgene (Daegu, Korea). Dulbecco’s modified phosphate buffered saline (PBS), Dulbecco’s modified eagle’s medium (DMEM), fetal bovine serum (FBS) were obtained from Hyclone (Logan, UT, USA). Glycerol, trimethylamine *N*-oxide (TMAO) and sucrose were obtained from Sigma-Aldrich (Yongin, Korea). Instruments used in the present study are Synergy HT plate reader (Biotek, Winooski, VT, USA), Nikon Eclipse T*i* microscope (Nikon, Tokyo, Japan).

**Table 3:** Percentage viability after 24 h of treatment at lower concentration of osmolytes. T98G cells were treated with osmolytes at 0.1 mM concentration with or without DBD plasma for 4 min. All values are means of three independent experiments.

Treatment	T98G (%)	HEK (%)
DBD Plasma (4 min)	31.2	61.3
Glycerol + Plasma	24.12	73.8
TMAO + Plasma	16	65.2
Sucrose + Plasma	23.61	71.7
Glycerol	95.43	96.6
TMAO	82	87
Sucrose	88.0	91.5

### 3.2. Osmolytes

We used glycerol, trimethylamine N-oxide (TMAO) and sucrose as osmolytes. These osmolytes were purchased from Sigma Aldrich (Yongin, Korea) are of high purity, were used without further purification. We performed all experiments at 25 mM, 50 mM and 75 mM concentrations.

### 3.3. Human Cell Culture

T98G malignant (brain cancer) and Human Embryonic Kidney (HEK) non-malignant cells were used in the present studies were purchased from Korean Cell Line Bank (KCLB), Seoul, Korea. These cell lines were cultured in 75 cm^2^ culture flasks (SPL, Pocheon-si, Korea) using Dulbecco’s modified Eagle’s medium (DMEM) supplemented with 10% fetal bovine serum, 1% nonessential amino acids, 1% glutamine, penicillin (100 IU/mL) and streptomycin (100 µg/mL) (all from Hyclone) or by distributor instructions. All cultures were maintained at 37 °C, 95% relative humidity and 5% CO_2_. Prior to each cytotoxicity test, the cells were harvested using trypsin–ethylenediaminetetraacetic acid (EDTA)–PBS solution (with 0.25% trypsin according to the distributor’s instructions) and diluted at a density of 10^5^ cells/mL for assays. Stock cultures were passaged every third day after harvesting the cells with 0.05% trypsin and seeding 8 × 10^3^ cells/cm^2^ in tissue culture flasks to maintain the cells in the exponential phase. All experiments were carried out in exponentially growing cells. The cell suspension was seeded into 24-well plates (SPL) at 100 µL/well, and incubated for approximately 20–24 h before tests in order to reach confluency. We performed a MTT assay and an analysis of the morphology of the cells treated by DBD plasma and osmolytes.

### 3.4. *In Vitro* Metabolic Viability Assay

Cells were seeded in 96-well plates at a concentration of 2–4 × 10^3^ cells/well in 200 μL of complete media and were incubated for 24 h at 37 °C in 5% CO_2_ atmosphere to allow for cell adhesion. All assays were performed in three independent sets of tests. A control group with no plasma treatment was run in each assay. Cell culture medium was changed and osmolytes are added with fresh media to the culture before plasma application. After 24, 48 and 72 h incubation of exposed cells to a plasma in the presence of osmolytes, each plate was carefully rinsed with 3 mL PBS buffer. Cytotoxicity was assessed using MTT (3-[4,5-dimethylthiazol-2yl]-2,5-diphenyltetrazolium bromide). MTT solutions, 250 μL (5 mg·mL^−1^ dd H_2_O), along with 4 mL of fresh and complete media, were added to each plate, and plates were incubated for 4 h. Following incubation, the media were removed, and the purple formazan precipitates in each well was sterilized in 2 mL dimethyl sulphoxide (DMSO). The absorbance was measured using a microplate reader at 540 nm, and the results were expressed as percentage (%) viability, which is directly proportional to metabolic active cell number [29,30,31,32]. Percentage (%) viability were calculated as follows:

% Viability = optical density in sample well/optical density in control well × 100

### 3.5. Statistical Analysis

Data have been expressed as means ± SD. Statistical analysis was done by student T-tests. Results were considered significant when *p* < 0.05.

## 4. Conclusions

We found that the effect of a 240 s plasma exposure and osmolytes at higher concentrations (25–75 mM) showed a inhibitory effect (decreased viability) in T98G cancer cells, which was almost similar to that of the plasma treated control. However plasma with lower concentration (0.1–1 mM) showed significant inhibitory effect on T98G cells, which was more than that of plasma treated control. Osmolytes had a cytoprotective effect on HEK normal cells specifically at lower concentration (0.1–1 mM), and we found that HEK cells could cope with the effect of plasma treatment only in the presence of lower concentratione of osmolytes after all incubation timee, showing enhanced viability, whereas osmolytes on T98G cells had no cytoprotective effect, showing a inhibitory effect with or without plasma treatment.

This treatment strategy shows a specific inhibitory effect of a 240 s plasma exposure in the presence of osmolytes against T98G brain cancer cells only, not on HEK normal cells. However further study against different cancer and normal cell line is going on with different plasma parametere/frequencies and will be reported in due course. Future work will also involve investigating the mechanism by which osmolytes have a protective effect in HEK normal cells and an inhibitory effect in T98G cells. Ongoing studies are directed at establishing whether the effects of a 240 s plasma exposure in the presence of osmolytes are through specific stress created by specific uptake of osmolytes by a specific cell line or through the changed nature of the osmolyte molecules caused by the plasma. 
